# Medición del contenido de aluminio en especímenes biológicos: aplicación en el laboratorio clínico

**DOI:** 10.1515/almed-2022-0014

**Published:** 2022-06-17

**Authors:** Sonia Pérez San Martín, Josep Miquel Bauçà, Eduardo Martínez-Morillo

**Affiliations:** Comisión de Elementos traza, Sociedad Española de Medicina de Laboratorio, Barcelona, España; Servicio de Análisis Clínicos, Hospital Universitario de Cabueñes, Gijón, España; Servicio de Análisis Clínicos, Hospital Universitari Son Espases, Palma de Mallorca, España; Servicio de Análisis Clínicos y Bioquímica Clínica, Complejo Asistencial Universitario de Salamanca, Salamanca, España

**Keywords:** absorción atómica, elementos traza, espectrometría de masas de plasma acoplado inductivamente, metales tóxicos, toxicidad

## Abstract

El aluminio se incorpora en el organismo principalmente por la dieta o la exposición ocupacional y se excreta por vía renal. Puede retenerse y presentar toxicidad especialmente en individuos con insuficiencia renal, incluso en aquellos que están sometidos a procesos de diálisis. Sus mecanismos de toxicidad están relacionados con el aumento del estrés oxidativo e inflamatorio, dishomeostasis del hierro y el calcio o la desregulación colinérgica, entre otros. En este documento se revisan los especímenes y métodos analíticos para la medida de aluminio en especímenes biológicos y agua de diálisis. Se detallan aquellos aspectos más relevantes para asegurar su calidad. Pretende ser una guía práctica para el desarrollo e implementación de un procedimiento de medida fiable del aluminio en un laboratorio clínico.

El aluminio en suero es el principal biomarcador para el estudio de su toxicidad, mientras que para la evaluación de una exposición crónica se recomienda la orina. Actualmente, la tecnología de espectrometría de masas de plasma acoplado inductivamente (ICP-MS) es la recomendada por presentar mejores límites de cuantificación, selectividad y robustez.

Se ofrecen recomendaciones claras sobre los especímenes utilizados en la medición de aluminio y las principales consideraciones preanalíticas, analíticas y postanalíticas.

## Introducción

El aluminio es el metal más abundante en la corteza terrestre, pero no se le conoce hasta la fecha ningún papel biológico en el ser humano. La Organización Mundial de la Salud (OMS) estableció una ingesta diaria tolerable de aluminio de 1 mg/kg de peso corporal, pero es ampliamente utilizado en la industria y como aditivo en agua de consumo, alimentos procesados, fórmulas infantiles y parenterales, desodorantes y medicamentos [1].

La exposición a altas concentraciones de aluminio, que se produce principalmente a través de la dieta y, en menor medida, del medio ambiente y de la exposición ocupacional, puede causar efectos perjudiciales a la salud. La vía inhalatoria ha demostrado ser altamente efectiva para la incorporación de aluminio en el organismo [2, 3].

Aunque la ingesta total de aluminio varía considerablemente según el lugar de residencia y la composición de la dieta, aproximadamente 10 mg de aluminio son ingeridos diariamente por el cuerpo humano [4]. En sangre, el aluminio se encuentra unido en más de un 80% a la transferrina y en individuos sanos es rápidamente excretado por los riñones [4, 5]. Puede producirse una acumulación de este metal en pacientes que reciben un gran aporte o aquellos con dificultades en su excreción [6], como en pacientes con enfermedad renal crónica (ERC) o sometidos a hemodiálisis, bien por la exposición al aluminio en el líquido de diálisis o por la ingestión de quelantes de fosfato que contienen aluminio y antiácidos. Hace varias décadas, la encefalopatía dialítica por aluminio asociada a osteomalacia y anemia era la principal complicación de la ERC en pacientes dializados [7, 8]. Actualmente, la última guía *Kidney Disease: Improving Global Outcomes* (KDIGO) recomienda evitar el uso a largo plazo de quelantes de fosfato que contengan aluminio en pacientes con ERC categoría G3-G5D y evitar la contaminación con aluminio del líquido de diálisis, especialmente en pacientes con ERC categoría G5D [9].

Asimismo, la Sociedad Española de Nefrología (SEN) recomienda que los pacientes en hemodiálisis mantengan niveles de aluminio en sangre inferiores a 20 µg/L (0,74 µmol/L) [10]. Sin embargo, dada la baja prevalencia actual de casos de intoxicación por aluminio en pacientes dializados, ciertos autores propugnan que su monitorización no es coste-efectiva en la mayoría de los pacientes dializados y otros recomiendan no realizarlo en pacientes sin factores de riesgo [7, 11].

La solubilidad de los compuestos de aluminio condiciona su toxicocinética y los riesgos para la salud [2]. El aluminio se acumula principalmente en los huesos, donde dificulta el intercambio de calcio, limitando el proceso de resorción ósea dependiente de la hormona paratiroidea (PTH) y la 1,25-dihidroxivitamina D y reduciendo la actividad de los osteoblastos [12]. Además de la acumulación del aluminio en tejido óseo en pacientes dializados, se ha observado también su acumulación en pacientes con enfermedad celíaca, hemocromatosis, anemia falciforme, exostosis, así como en pacientes con prótesis de rodilla o cadera [13]. Esta acumulación en tejido óseo puede ser especialmente relevante en niños y adolescentes, con importantes consecuencias clínicas [14].

Recientemente, se han detectado niveles elevados de aluminio en tejido cerebral en pacientes con enfermedad de Alzheimer (AD), autismo y epilepsia [1, 15]. El aluminio induce daño cerebral y alteraciones del comportamiento, a través de mecanismos no totalmente esclarecidos como la apoptosis, la fosforilación de la proteína tau, acumulación de la proteína β-amiloide, formación de especies reactivas de oxígeno, la necrosis de células neuronales, la dishomeostasis de metales y cambios en el sistema de defensa antioxidante [16, 17], que da lugar a la apoptosis de las neuronas y las células gliales [18] y desencadena un estado proinflamatorio que puede asociarse a un aumento de eventos vasculares [17].

En un metanálisis, Wang et al. [19], concluyeron que la exposición crónica al aluminio se encontraría asociada con un mayor riesgo de AD, mientras que Colomina et al. [20], concluyeron que dicha asociación es plausible, aunque aún no se ha establecido la concentración de aluminio, el tiempo de exposición u otros factores de riesgo necesarios.

Con respecto al uso del aluminio en las vacunas, aunque ciertos autores afirman que este elemento puede causar mialgia persistente, fatiga o enfermedad autoinmune, no se ha podido establecer una asociación entre el aluminio y estas enfermedades [21]. Las sales de aluminio se usan como adyuvantes desde hace décadas para aumentar el efecto inmunológico y, en algunas, para asegurar la efectividad. Los Centros para el Control y la Prevención de Enfermedades (CDC) consideran que la cantidad de aluminio utilizada es baja y que el cuerpo no la absorbe fácilmente [22], presentando mínimas reacciones adversas en la zona de inyección [1].

En la actualidad, el laboratorio clínico realiza la medida de la concentración de aluminio principalmente para la monitorización de pacientes sometidos a diálisis y del propio agua de diálisis y para el control del desgaste de implantes protésicos metálicos.

## Objeto y campo de aplicación

El objeto de este documento es revisar los métodos analíticos para la medida del contenido de aluminio en especímenes biológicos y sus implicaciones fisiopatológicas, además de ofrecer una guía práctica para implementar esta tecnología en el laboratorio clínico. Es de aplicación en los laboratorios clínicos con instalaciones y equipamiento para el estudio de elementos traza y metales tóxicos.

## Especímenes biológicos para la medición de aluminio

### Consideraciones generales

La contaminación es el principal problema en la medición de elementos traza, incluido el aluminio. Hay que tener especial cuidado en la obtención y manipulación de las muestras. En la guía de *Clinical and Laboratory Standards Institute* (CLSI) se detallan los procedimientos que deben tenerse en cuenta en la fase preanalítica [23].

La Sociedad Española de Medicina de Laboratorio (SEQC^ML^) publicó los requerimientos técnicos y ambientales y las medidas de seguridad que se deben tener en cuenta en los laboratorios clínicos para la determinación de elementos traza [24, 25]. Además, es necesario la utilización de patrones y reactivos de alto grado de pureza y la utilización de agua ultrapura tipo I [23].

### Sangre

La medición de la concentración de aluminio en suero es útil para evaluar una posible intoxicación. Es la prueba de elección para el cribado rutinario y evaluar el desgaste de implantes metálicos protésicos [3, 26, 27].

La mayoría de los tubos y utensilios para la extracción de sangre contienen gomas hechas de silicato de aluminio. Se recomienda el empleo de suero obtenido en tubos de vacío específicos para elementos traza. No interfiere el uso de tubos con gel separador, pero sí debe evitarse el empleo de tubos de vidrio [23]. Es responsabilidad de cada laboratorio asegurar la intercambiabilidad de resultados, de acuerdo con las especificaciones de calidad.

Se debe comprobar que el material que va a estar en contacto con el espécimen (tubos de ensayo, puntas de pipeta, contenedores, etc.) no presenta posibilidad de contaminación con aluminio. En caso contrario, debe lavarse previamente, dejándolo entre 12 y 24 horas en una solución de ácido nítrico al 10% y enjuagar varias veces con agua desionizada antes de su uso [23].

Una vez extraída la sangre y dejado que coagule, se centrifuga durante 10 minutos a 1.000–1.200 g sin destapar para evitar una contaminación procedente de la centrífuga o evaporaciones [23]. El suero puede conservarse en tubos de polipropileno o poliestireno bien tapados durante 15 días a 4 °C, o congelarse hasta su procesamiento [28].

### Orina

La orina es la muestra de elección para monitorizar la exposición crónica a aluminio y para evaluar una exposición crónica, como por ejemplo exposiciones laborales o ambientales, al ofrecer una mayor sensibilidad [3, 26, 27].

Se ha comprobado que, en trabajadores expuestos al aluminio, la concentración urinaria de aluminio tras 1 o 2 días sin exposición es un buen indicador de la cantidad de aluminio almacenada en el organismo [27].

Para su medición, es preferible recoger orina de 24 horas. Es muy importante obtener un valor preciso de diuresis para la correcta valoración de la concentración de aluminio en la orina de 24 horas. Análogamente al suero, se recomienda que el material empleado para el estudio de la orina sea estudiado en términos de posible contaminación por aluminio y, si no es así, lavado con ácido previamente. A fin de mantener la estabilidad de las muestras, éstas se podrían acidificar al recepcionarlas en el laboratorio [23]. Asimismo, es esencial evitar los errores en la recogida de la orina de 24 horas, así como la contaminación del contenedor con aluminio exógeno.

Una vez homogeneizadas la muestras, se alicuotan en tubos limpios y conservan refrigeradas o congeladas hasta su procesamiento. Si se han sometido a congelación, tras descongelarlas es necesario una buena homogeneización y centrifugación.

### Hueso

La muestra ósea se extrae normalmente de la cresta ilíaca con un trocar, por biopsia intraoperatoria, o bien *post mortem* en el caso de las muestras obtenidas para los estudios del contenido de aluminio de la población de referencia [29]. Se introducen en un envase de polipropileno y se almacenan a −20 °C hasta su procesamiento de digestión. El material óseo es estable 3 meses a −20 °C y liofilizado un año [28].

### Agua y líquido de diálisis

Han de seguirse las mismas recomendaciones en cuanto a la recogida y manipulación que para el suero y la orina [28]. Pueden recogerse las muestras de agua en diferentes tubos estériles como los que se utilizan para suero, o alternativamente en un bote de orina si van a realizarse determinaciones adicionales que requieran volúmenes mayores.

La guía de la SEN recomienda que la concentración máxima de aluminio en el agua ultrapura para hemodiálisis sea de 10 µg/L (0,37 µmol/L), siendo su control semestral [10].

El estudio de la concentración de aluminio en pelo, uñas u otros líquidos biológicos está fuera del alcance de estas recomendaciones [30], [31], [32].

## Metodología para la medición de aluminio

Hay múltiples técnicas analíticas para cuantificar aluminio, aunque algunas de ellas están totalmente en desuso debido a unos límites de detección insuficientes o la considerable cantidad de interferencias existentes, como son la espectroscopía de absorción molecular ultravioleta-visible y de fluorescencia molecular, la espectrometría de absorción atómica de llama, la fluorescencia de rayos X y el análisis por activación de neutrones. Estas técnicas no son objeto de esta revisión.

### Espectrometría de absorción atómica por atomización electrotérmica

La espectrometría de absorción atómica por atomización electrotérmica (ETAAS) ha sido históricamente la técnica de elección en los laboratorios clínicos debido a las ventajas de su manejo sencillo, no necesitar pretratamiento de la muestra y emplear pequeños volúmenes de muestra. El límite de detección se encuentra en torno a 1–2 µg/L (0,04–0,07 µmol/L) [28].

Esta técnica requiere del uso de correctores de fondo, siendo el de elección el corrector con efecto Zeeman, y evitando las interferencias moleculares, lo que le confiere a la técnica una elevada especificidad. También se emplean modificadores de matriz, que permiten el desplazamiento de los átomos de cloro y previenen la formación de cloruro de aluminio volátil en las etapas previas a la atomización. En la práctica, se suelen diluir los especímenes con agua, ácido nítrico y un surfactante, como el Tritón X-100^®^. También puede emplearse nitrato de magnesio como modificador, con el fin de de reducir la volatilidad del cloruro de aluminio. No obstante, los modificadores de matriz pueden ser una fuente de contaminación y se aconseja reducir su uso siempre que sea posible [28].

El procedimiento de medida de la concentración de aluminio en distintos tipos de muestra mediante ETAAS se describe en el Material Suplementario.

### Espectrometría de masas con plasma de acoplamiento inductivo

Su uso para la cuantificación de elementos traza y metales tóxicos ha ido creciendo de manera exponencial en matrices biológicas, a pesar de la complejidad y el elevado coste del analizador. En el año 2021, la espectrometría de masas con plasma de acomplamiento inductivo (ICP-MS) fue la técnica más utilizada en Europa, de acuerdo con los datos del programa de garantía externa de la calidad *Occupational and Environmental Laboratory Medicine* (OELM) para aluminio sérico.

Además de la mejora en los límites de detección y la selectividad, la principal ventaja de estos analizadores es la posibilidad de análisis multielemental y la sencillez en la preparación de la muestra [33], siendo la técnica de elección cuando se requiere un análisis de múltiples elementos y con un número elevado de muestras [34].

Las elevadas temperaturas que alcanza el plasma (6.000–10.000 K), la estabilidad y el entorno de argón químicamente inerte eliminan muchas de las interferencias presentes en otros tipos de espectroscopia atómica, pudiendo alcanzar límites de detección cercanos a 0,1-1 µg/L (0,004–0,04 µmol/L) para la mayoría de los elementos de la tabla periódica. Por ello, el ICP-MS es la tecnología recomendada para la determinación de aluminio en especímenes biológicos y aguda de diálisis.

Dada la elevada sensibilidad del equipo, y debido a que una mínima variación en la preparación o introducción de la muestra puede conllevar un cambio significativo en la respuesta instrumental, se aconseja el uso de patrones internos, cantidad conocida de un compuesto diferente del analito y ausente en la muestra, y que se añade a ésta, para la corrección de las fluctuaciones típicas de la respuesta instrumental [33].

Existen dos tipos de interferencias en ICP-MS [27, 33]:

– Espectroscópicas: surgen cuando una especie iónica tiene una relación masa/carga (m/z) igual a la del analito. Suelen provenir de iones poliatómicos y de iones de óxidos refractarios. Estas interferencias se evitan seleccionando un isótopo diferente del elemento traza a analizar, que presente una relación m/z diferente a la de la sustancia interferente o bien utilizando una celda de colisión con un gas inerte (eliminación cinética) o reactivo (eliminación química).
– No espectroscópicas: debidas a la matriz, que suele provocar un cambio de la señal por una alteración en el transporte de la muestra, en el grado de ionización del analito en la fuente de plasma, o en el grado de extracción de los iones. Pueden corregirse diluyendo la muestra, separando previamente los compuestos interferentes, introduciendo la muestra de forma diferente (por vaporización electrotérmica, uso del nebulizador a 2 °C, por inyección de flujo), o bien empleando un patrón interno. En algunos casos, puede ser necesario realizar la cuantificación del analito por el método de adición de estándar.

#### Procedimiento de medida de la concentración de aluminio en distintos tipos de muestra mediante ICP-MS

##### Condiciones analíticas

Las condiciones analíticas dependen del equipo utilizado. Se monitoriza el isótopo de aluminio de masa atómica 27 (único isótopo natural y estable de este elemento), utilizando escandio (Sc) como patrón interno, que tiene una masa atómica de 44,95.

##### Preparación de las muestras

Dados los bajos límites de cuantificación de esta tecnología, pueden realizarse diluciones mayores de las muestras. Se obtienen resultados satisfactorios utilizando diluciones cercanas a 1/14^o^ 1/18 para el suero y de 1/9 para la orina con HNO_3_ 0,5%, para los patrones y los controles. En función del volumen de pipeteo del analizador, se puede recomendar una dilución al 1/14, mezclando 250 µL de muestra con 3,250 µL de HNO_3_ 0,5% como diluyente.

##### Preparación de patrones

A partir de un patrón certificado o de una disolución de un patrón primario de 1 g/L de Al (37,03 mmol/L) se prepara una disolución acuosa de almacenaje de 100 mg/L (3,7 mmol/L) y por diluciones seriadas, los patrones de la curva de calibración. Dado que concentraciones séricas de aluminio superiores a 60 µg/L (2,22 µmol/L) sugieren una posible contaminación y son poco frecuentes [27], no es necesario que la recta de calibrado supere ese valor. Si se decide trabajar con muestras diluidas al 1/14, se puede hacer una recta utilizando calibradores de concentración comprendida entre 0,5 y 4,5 µg/L, que permitiría cuantificar muestras desde 7 a 63 µg/L (0,26 a 2,33 µmol/L) de aluminio. En cambio, si se trabaja con una menor dilución como 1/6, pueden cuantificarse muestras con concentraciones a partir de 3 µg/L (0,11 µmol/L).

De forma similar al resto de tecnologías analíticas, si se detecta efecto matriz en la cuantificación de aluminio, se recomienda realizar una calibración por adición de estándar.

### Aseguramiento de la calidad

#### Especificaciones de la calidad analítica basadas en la variabilidad biológica

Los criterios de variabilidad biológica no pueden aplicarse adecuadamente en el caso del aluminio, al ser un elemento tóxico sin función biológica conocida, para establecer recomendaciones de calidad analítica, ya que los datos son muy escasos [35].

Taylor et al. recomendaron para el aluminio un error total inferior a 5 µg/L (o menos del 20%) del valor diana, siendo este el objetivo de calidad analítica que se ha de tratar de cumplir [36].

#### Materiales de control

La incorporación de materiales de referencia al proceso analítico es básica para la evaluación de la calidad y tiene un papel principal en la trazabilidad de las mediciones. Los laboratorios deben utilizar, en su trabajo diario, controles de calidad internos con matriz igual o similar a la de los especímenes a analizar (suero, sangre, orina) a fin de asegurar la fiabilidad de sus resultados. Por ello, para suero se recomiendan los controles Lyphochek^®^ Whole Blood Metals Quality Control (BIO-RAD, QCNetT™), Seronorm Trace Elements Serum (Seronorm™ Trace Element Serum [Nycomed Pharma AS, Noruega]), ClinChek^®^ Serum Controls, lyophilised for Trace Elements (RECIPE^®^) u otros similares.

Para muestras de orina se sugieren los siguientes: Lyphochek^®^ Urine Metals Control (BIO-RAD), Seronorm Trace Elements Urine (Nycomed), ClinChek^®^ Urine Controls, lyophilised for Trace Elements (RECIPE^®^) u otros similares.

#### Programas de evaluación externa de la calidad

Asimismo, es conveniente la participación en Programas de Garantía Externa de la Calidad (EQA) de intercomparación de laboratorios, con el fin de asegurar veracidad, la precisión y la reproducibilidad de sus resultados, además de ser un requisito de la Norma UNE-EN ISO 15189:2013, exigido por la Entidad Nacional de Acreditación (ENAC) a los laboratorios clínicos acreditados.

Existen numerosos programas externos de evaluación de la calidad específicamente diseñados para elementos traza que incluyen el aluminio.

La SEQC^ML^ participa en la organización del Programa de Garantía Externa de OELM junto con otros países europeos, compartiendo las muestras, la base de datos y el diseño de los informes con un enfoque federativo, aunque se mantienen como organizaciones independientes con sus propias inscripciones al programa, idioma, reuniones de usuarios y otros sistemas de soporte (http://www.trace-elements.eu). Las matrices que presentan son suero, sangre total y orina.

Otros programas que destacar son el UK NEQAS for Trace Elements (TEQAS, Reino Unido), que cuenta con matrices de suero, sangre, orina y agua y fluidos de diálisis y el QMEQAS, del *Centre de Toxicologie du Québec* (Canadá), que dispone de las matrices suero, sangre, orina y pelo.

## Intervalos de referencia

Las concentraciones de aluminio pueden variar significativamente dentro de una población y entre regiones geográficas diferentes, pudiendo esto depender de los hábitos alimentarios, el entorno laboral, el estilo de vida y las variaciones genéticas [27, 37].

En Australia, en un estudio reciente con 120 individuos adultos sanos, observaron que las concentraciones medias plasmáticas de aluminio, medidas por ICP-MS, fueron de 6,9 μg/L, no encontrándose diferencias significativas por edad o sexo [37]. Datos similares a estos se obtuvieron en el estudio francés IMEPOGE, con 2000 participantes sanos, en el que se observó que la concentración media de aluminio fue de 4,32 μg/L, con valores significativamente mayores en hombres que en mujeres [38]. En otros dos estudios, en Bélgica y en Canadá, la mediana de las concentraciones de aluminio fue inferior al límite de detección de la técnica (3,1 y 8 μg/L, respectivamente) [39, 40].

Con respecto a las concentraciones de aluminio en orina, en el estudio francés se obtuvieron unos valores medios de 4,25 μg/L o de 3,99 μg/g creatinina [38] y en un estudio belga, la mediana de los niveles de aluminio fue de 2,17 μg/L o de 2,04 μg/g creatinina [41].

Para el contenido del aluminio en el hueso, que representa el depósito preferencial cuando existe una sobrecarga, la literatura muestra una gran dispersión de los datos, con valores medios desde 1 hasta 9 µg/g de peso seco [27].

Cada laboratorio debe establecer y verificar sus propios valores de referencia mediante un procedimiento estandarizado [42] o, en su defecto, verificar los valores de referencia adoptados. Destacada la importancia de establecer y revisar periódicamente los valores de referencia con el fin de garantizar y asegurar la calidad de los resultados ofrecidos, se proponen únicamente con una finalidad orientativa, los siguientes valores de referencia [23, 27]:

– Suero: <10 μg/L (<0,37 µmol/L)
– Orina: <7 μg/día (<0,26 µmol/día)
– Tejido: <2 μg/g peso seco (<0,07 µmol/kg)

Adicionalmente, la Clínica Mayo ha publicado recientemente los siguientes valores de referencia en adultos [43]:

– Suero: <7 μg/L (0,26 µmol/L), <60 μg/L (2,23 µmol/L) (en pacientes dializados).

Pacientes con un implante protésico metálico con aluminio pueden tener concentraciones superiores a 6 μg/L. Una elevación moderada de la concentración (6–10 μg/L) sugiere que la prótesis se encuentra en buenas condiciones. Concentraciones de aluminio superiores a 10 μg/L (0,37 µmol/L) sugieren un desgaste de la prótesis.

– Orina 24 h: <13 μg/24 h (<0,48 µmol/24 h)
– Orina micción aislada: <14 μg/g creatinina (0,52 µmol/g creatinina)

## Conclusiones

En este artículo se revisan los especímenes y métodos analíticos para la medida de aluminio en especímenes biológicos y agua de diálisis; con el objetivo de ser utilizado como una guía básica para cualquier profesional del laboratorio clínico que desee introducir la cuantificación de aluminio en su centro de trabajo. Además de las consideraciones preanalíticas, se ofrecen recomendaciones para el aseguramiento de la calidad, así como para el establecimiento de intervalos de referencia y la interpretación de resultados.

## Supplementary Material

Supplementary Material

## Material Suplementario

La versión en línea de este artículo ofrece material suplementario (https://doi.org/10.1515/almed-2022-0014).
