# Supplementary material for: Medición del contenido de aluminio en especímenes biológicos: aplicación en el laboratorio clínico
Source: Adv Lab Med. 2022 Jun 17;3(2):160–6. [Article in Spanish] doi: 10.1515/almed-2022-0014 (PMC10197762; doi:10.1515/almed-2022-0014)
Supplement: Supplementary file 1 — Supplementary Material [file j_almed-2022-0014_suppl.docx]

**ANEXO 1. MATERIAL SUPLEMENTARIO 1**

Procedimiento de medida de la concentración de aluminio en distintos tipos de muestra mediante *e*spectrometría de absorción atómica por atomización electrotérmica.

#### **Condiciones analíticas**

Se ha de emplear una lámpara de cátodo hueco o de descarga monoelemental para obtener la mayor intensidad de señal luminosa, la longitud de onda de 309,3 nm y la rendija de 0,7 nm. Las cámaras de grafito han de ser pirolíticas con plataforma tipo L’Vov. En general, los métodos descritos emplean tres etapas de secado para evaporar la muestra (Tabla 1, Tabla 2). Las temperaturas de secado están en el rango de 90-200 ºC, las de mineralización entre 600 y 1500 ºC y las de atomización en torno a 2600 ºC. Un programa rápido y al alcance de cualquier equipo para suero/plasma es el representado en la Tabla 1 y para orina y hueso el representado en la Tabla 2. No obstante, cada usuario deberá optimizar estas condiciones de acuerdo con su instrumentación (48, 49). Se recomienda utilizar un volumen de muestra de 20 μL. Se emplea lectura en área de pico con un tiempo de integración de 2 s. Se recomienda un paso adicional de limpieza a 2650 ºC.

# Con esta técnica se alcanzan límites de cuantificación cercanos a 0,14 µmol/L.

#### **Preparación de la muestra**

Para la medida de la concentración de aluminio en suero se recomienda la dilución de las muestras de suero en proporción 1:2 ó 1:3 con el modificador de matriz conteniendo ácido nítrico (HNO_3_) al 0.2% y Triton X-100®.

Los especímenes de la orina acidificados se preparan en el momento de ser analizados, se centrifugan y se diluyen 1:2 con un modificador conteniendo ácido nítrico al 0,1 % y Triton X-100 al 0,2 % como surfactante.

Todo el proceso preparativo para la medición de aluminio en hueso se realiza en una cámara limpia para evitar contaminaciones del polvo atmosférico. Ocho horas antes del procesamiento en el horno de grafito se procede al secado del cilindro óseo (20-30 mg de peso) en la estufa a 90 ºC hasta conseguir un peso constante. A continuación, en recipientes de teflón de 3 mL, se procede a la digestión con 200 µL de ácido nítrico calidad suprapur y se mantiene una hora a la temperatura ambiente y cinco horas en idénticas condiciones en estufa a 85 – 90ºC. Se obtiene un líquido claro y transparente, que se enrasa hasta 500 µL con agua. Esta disolución se diluye a su vez de una forma adecuada con agua tratada por ósmosis inversa. Las diluciones según los contenidos en aluminio de las muestras, ya que en las condiciones descritas la medida es buena para los intervalos de 10 a 40 µg/L (0,37 a 1,48 µmol/L).

Para la medición en el agua de la red y los líquidos de diálisis, los especímenes se diluyen 1:3 con ácido nítrico al 0,2 %. Pueden realizarse diluciones superiores si las concentraciones son elevadas.

#### **Preparación de los patrones**

Se pueden preparar patrones de concentración 10, 20 y 40 µg/L (0,37, 0,74 y 1,48 µmol/L), en HNO_3_ al 0,2%. Se puede partir de un patrón certificado de 1 g/L en HNO_3_ al 2%. El blanco está constituido únicamente por HNO_3_ al 0,2% y Triton X-100® al 0,2%.

En el caso del hueso, la calibración se realiza mediante adiciones estándar que, aunque laborioso, es el sistema que mejor consigue minimizar el efecto matriz dado el elevado contenido en fosfatos de la disolución procedente de la digestión ósea *(*50*)*.

Tabla 1. Programa de temperaturas recomendado para el análisis de aluminio en suero por ETAAS

| Etapa | Temperatura (ºC) | Tiempo (s) | | Flujo de argon (mL/min) | Lectura |
| --- | --- | --- | --- | --- | --- |
|  |  | Rampa inicial | Rampa final |  |  |
| Secado 1 | 90 | 5 | 5 | 300 | No |
| Secado 2 | 130 | 10 | 10 | 300 | No |
| Secado 3 | 200 | 5 | 5 | 300 | No |
| Mineralización 1 | 600 | 1 | 20 | 300 | No |
| Mineralización 2 | 1.500 | 10 | 15 | 300 | No |
| Atomización | 2.600 | 0 | 2 | 0 | Sí |
| Limpieza | 2.650 | 1 | 2 | 300 | No |

Tabla 2. Programa de temperaturas recomendado para el análisis de aluminio en orina y en hueso por ETAAS

| Etapa | Temperatura (ºC) | Tiempo (s) | | Flujo de argon (mL/min) | Lectura |
| --- | --- | --- | --- | --- | --- |
|  |  | Rampa inicial | Rampa final |  |  |
| Secado 1 | 90 | 5 | 5 | 300 | No |
| Secado 2 | 130 | 5 | 5 | 300 | No |
| Secado 3 | 200 | 2 | 2 | 300 | No |
| Mineralización 1 | 600 | 1 | 20 | 300 | No |
| Mineralización 2 | 1.450 | 2 | 15 | 300 | No |
| Atomización | 2.600 | 0 | 2 | 0 | Sí |
| Limpieza | 2.650 | 1 | 2 | 300 | No |
